# Prognostic value of CD8^+^T cells related genes and exhaustion regulation of Notch signaling pathway in hepatocellular carcinoma

**DOI:** 10.3389/fimmu.2024.1375864

**Published:** 2024-04-08

**Authors:** Qing Pu, Lihua Yu, Xiaoli Liu, Huiwen Yan, Yuqing Xie, Xue Cai, Yuan Wu, Juan Du, Zhiyun Yang

**Affiliations:** ^1^ Center of Integrative Medicine, Beijing Ditan Hospital, Capital Medical University, Beijing, China; ^2^ School of Traditional Chinese Medicine, Capital Medical University, Beijing, China; ^3^ The First Clinical Medical College of Beijing University of Traditional Chinese Medicine, Beijing, China; ^4^ Beijing Key Laboratory of Emerging Infectious Diseases, Institute of Infectious Diseases, Beijing Ditan Hospital, Capital Medical University, Beijing, China; ^5^ Beijing Institute of Infectious Diseases, Beijing, China; ^6^ National Center for Infectious Diseases, Beijing Ditan Hospital, Capital Medical University, Beijing, China

**Keywords:** hepatocellular carcinoma, CD8^+^T cells exhaustion, risk scoring, prognosis, Notch signaling pathway

## Abstract

Immunotherapy has emerged as the primary treatment modality for patients with advanced Hepatocellular carcinoma (HCC). However, its clinical efficacy remains limited, benefiting only a subset of patients, while most exhibit immune tolerance and face a grim prognosis. The infiltration of immune cells plays a pivotal role in tumor initiation and progression. In this study, we conducted an analysis of immune cell infiltration patterns in HCC patients and observed a substantial proportion of CD8^+^T cells. Leveraging the weighted gene co-expression network analysis (WGCNA), we identified 235 genes associated with CD8^+^T cell and constructed a risk prediction model. In this model, HCC patients were stratified into a high-risk and low-risk group. Patients in the high-risk group exhibited a lower survival rate, predominantly presented with intermediate to advanced stages of cancer, displayed compromised immune function, showed limited responsiveness to immunotherapy, and demonstrated elevated expression levels of the Notch signaling pathway. Further examination of clinical samples demonstrated an upregulation of the Notch1^+^CD8^+^T cell exhaustion phenotype accompanied by impaired cytotoxicity and cytokine secretion functions that worsened with increasing Notch activation levels. Our study not only presents a prognostic model but also highlights the crucial involvement of the Notch pathway in CD8^+^T cell exhaustion—a potential target for future immunotherapeutic interventions.

## Introduction

Primary liver cancer (PLC) is a prevalent malignant tumor that poses challenges in early diagnosis. The majority of PLC patients are initially diagnosed at an advanced stage, resulting in missed opportunities for surgical treatment and high mortality rates (1). In the forthcoming two decades, it is anticipated that there will be a persistent escalation in both morbidity and mortality rates of PLC, thereby imposing an enormous global health burden (2, 3). Over 90% of PLC are hepatocellular carcinoma (HCC), predominantly attributed to chronic hepatitis B infection (4). The immune system plays a dual role in the occurrence and development of tumors, as it not only exerts anti-tumor effects by destroying tumor cells and inhibiting their proliferation but also facilitates tumor growth by selectively favoring tumor cells capable of adapting to the immune environment and shaping a microenvironment that fosters tumorigenesis (5). In the tumor microenvironment (TME), long-term, repeated chronic inflammation can promote angiogenesis and immune system suppression, which can affect the effectiveness of treatment (6). Previous research has consistently demonstrated that the density, composition, and function of immune cells infiltrating the tumor are closely associated with the therapeutic outcome (7).

In recent years, immunotherapy based on the regulation of the body’s immune system has made a breakthrough in the treatment of advanced HCC, and immune checkpoint inhibitors (ICIs) have replaced multi-kinase inhibitors as the preferred treatment for advanced HCC (8). Immunotherapy offers additional options for HCC and brings hope for the treatment of advanced patients. Despite these advancements, clinical outcomes reveal that only 15-20% of patients derive benefits from immunotherapy (9). Furthermore, it should be noted that a subset of patients who initially responded well to treatment eventually exhibited the development of tolerance (10). Hence, acquiring a thorough comprehension of the immune regulatory mechanism and precisely discerning the individuals who would derive advantages from immunotherapy is of paramount importance. Several studies have demonstrated a significant correlation between the infiltration of CD8^+^T cells and the prognosis of cancer (11, 12). The dysfunction of CD8^+^T cells hampers the anti-tumor immune function in patients, thereby affecting the effectiveness of treatment. Previously, our focus was primarily on examining the association between the quantity of CD8^+^T cells and the prognosis of HCC patients (13). Recognizing the crucial role of CD8^+^T cell function in immune response, this study aims to delve deeper into elucidating the intricate association between CD8^+^T cell function and prognosis.

**Figure 1:**
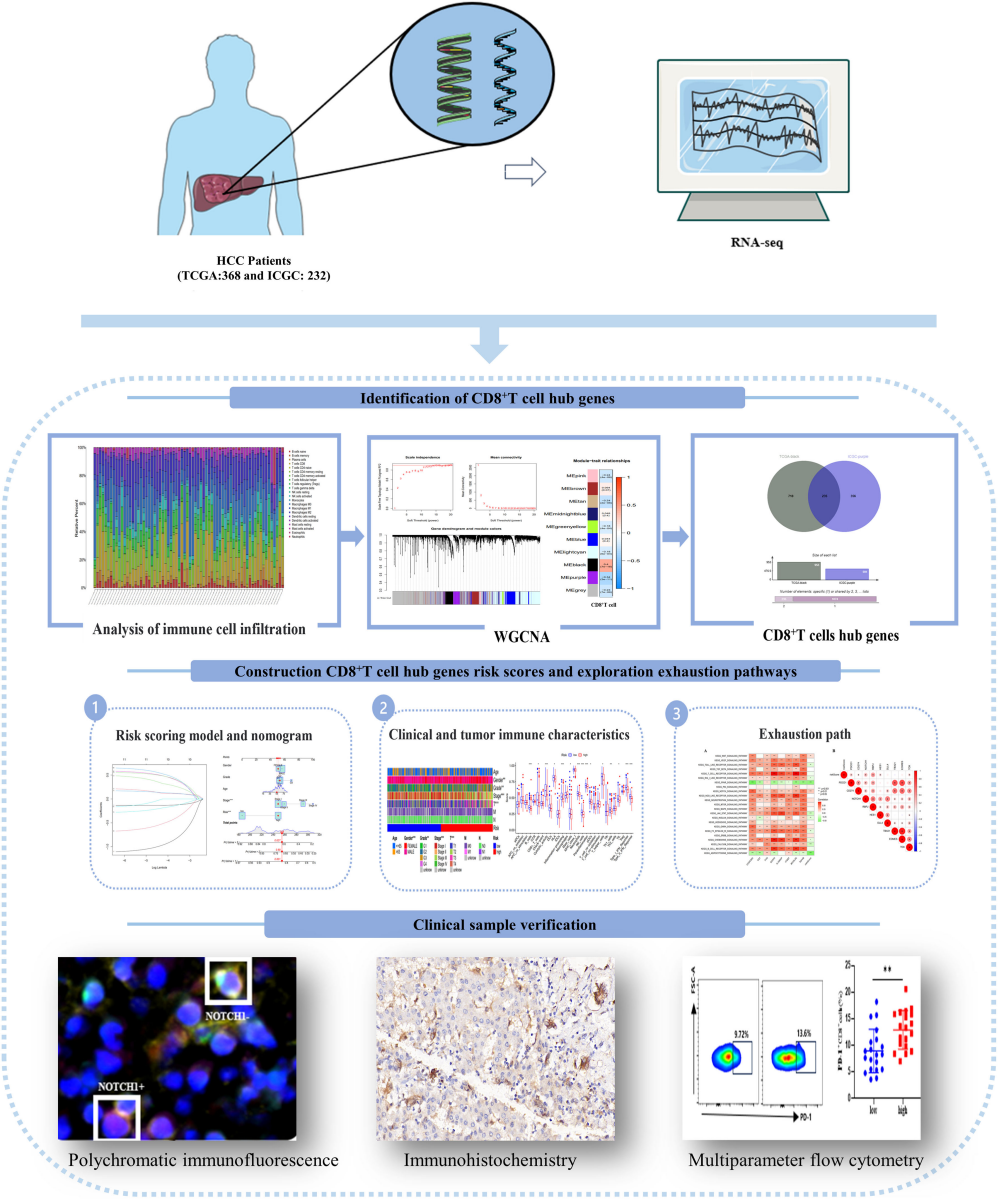
An article flow chart describing the process of experimental flow and data analysis.

In this study, we assessed the prognostic significance of CD8^+^T cell transcriptome data in HCC patients. Additionally, we developed a risk score model and nomogram based on the hub genes that regulate CD8^+^T cells. We evaluated the model’s ability to discriminate based on clinical features and immune function, and further investigated the pathway mechanism responsible for CD8^+^T cell exhaustion. To validate our findings, we conducted multicolor flow cytometry, immunohistochemistry, and immunofluorescence experiments using clinical HCC patient data. The technical approach employed in this study is illustrated in **Figure 1**.

## Materials and methods

### Patients and datasets

This study utilized RNA-seq (FPKM) data obtained from two datasets: the TCGA-LIHC dataset (n=368) from the Cancer Genome Atlas Program (TCGA, https://portal.gdc.cancer.gov/) database and the LIRI-JP dataset (n=232) from the International Cancer Genome Consortium (ICGC, https://dcc.icgc.org/) database. These datasets collectively comprised a total of 600 HCC patients. And there are multiple omics data and corresponding clinical information (**Supplementary Table 1**). In addition, the HCC patients used for results verification were hospitalized in Beijing Ditan Hospital, affiliated to Capital Medical University, from November 2021 to July 2022.

### Identification of CD8+T cells hub genes

To assess the infiltration of CD8^+^T cells in the tumor microenvironment (TME), we employed R software and CIBERSORT deconvolution algorithm (14) to estimate the relative abundance of 22 common immune infiltrating cell types in HCC samples. Subsequently, weighted gene co-expression network analysis (WGCNA) (15) was conducted on these results, aiming to investigate the gene modules associated with CD8^+^T cells. The gene spectrum data was initially normalized, and subsequently outliers were filtered out. The weighted value of correlation coefficients was subsequently calculated, ensuring that the connections between genes conform to the scale-free network distribution. The optimal power value (weight parameter) is determined based on soft threshold R^2^ and average connectivity. Adjacency matrix and topological overlap matrix (TOM matrix) are gradually established, and gene clustering and dynamic division of modules are carried out.

Based on the analysis results of WGCNA, the genes intersection of TCGA and ICGC with the largest correlation with CD8^+^T cells was selected as the hub genes of CD8^+^T cells. The molecular and pathway characteristics of hub genes were determined by the Gene Ontology (GO), the Kyoto Encyclopedia of Genes and Genomes (KEGG), and Protein-Protein Interaction Networks (PPI) constructed according to maximal clique centrality (MCC) scores.

### Construction of risk scoring model and nomogram

The risk score was constructed using the CD8^+^T cell hub gene set. The TCGA dataset was divided into a training set and a validation set in a 7:3 ratio, with the ICGC dataset used for external validation. The difference in survival time between the high and low risk groups was evaluated using the Kaplan-Meier (KM) curve. The model discrimination was assessed by calculating the Area under the curve (AUC) of the Receiver Operating Characteristic (ROC) curve at 1, 2, and 3 years and performing PCA analysis. A nomogram was constructed using univariate and multivariate cox analysis, combining the risk score with clinical indicators. Additionally, forest plot, 1-year and 3-year ROC curves, and calibration curves were drawn.

### Analysis of clinical features and tumor immune features of risk score subgroups

The study analyzed the clinical characteristics of the risk score subgroups and evaluated the differences in risk scores based on tumor stages and grades. Heatmaps were used to visualize these differences, as well as the differences in tumor stages and tumor grades within each risk score subgroup. Additionally, the study obtained immune data set (immune.gmt) from the MSigDB database and TCIA score files from the TCIA database (https://tcia.at/home) to examine the tumor immune characteristics. The analysis focused on identifying differences in immune cell function among the risk score subgroups and determining the advantageous groups for immunotherapies.

### Exhaustion pathway exploration and clinical sample collection

Gene Set Variation Analysis (GSVA) (16) was utilized to analyze the signaling pathways that play a significant role in high and low risk groups. The study aimed to explore the correlation between modeling genes, risk scores, and various KEGG signaling pathways, as well as the potential pathway mechanisms leading to the exhaustion of CD8^+^T cells. And it was verified in clinical HCC patients. Blood samples (39 cases in total), cancer tissues and adjacent tissues (30 cases in total) were collected from HCC patients. Informed consent was obtained from all patients prior to sample collection. The study was approved by the Ethics Committee of Beijing Ditan Hospital Affiliated to Capital Medical University (NO. DTEC-KY2019-009-05), and all methods and procedures were carried out in accordance with the guidelines.

### Multiparameter flow cytometry

Peripheral blood mononuclear cells (PBMC) were extracted from 4ml of venous blood obtained from HCC patients for flow cytometry analysis. We used the anti-human CD8 (Biolegend) marker to identify CD8^+^T cells. Notch1^+^CD8^+^T cells were identified using the anti-human Notch1 (BD) antibody. Additionally, we assessed the expression of co-inhibitory molecules on the cell surface using anti-human TIGIT (BD), anti-human TIM-3 (BD), anti-human CD39 (Biolegend), and anti-human PD-1 (BD) antibodies. Furthermore, we evaluated the killing function of the cells by detecting the expression of anti-human Perforin (Biolegend) and anti-human GranzymeB (BD) antibodies.

### Polychromatic immunofluorescence and immunohistochemistry

Paraffin sections of tumor tissues and adjacent tissues of patients with HCC were used for immunofluorescence staining. The paraffin sections were impregnated with xylene, gradient ethanol and ddH2O tablets successively, after which the antigen was repaired by microwave, followed by closure and subsequent incubation with primary and secondary antibodies. This was followed by color reaction, signal amplification, removal of the antibody complex, restaining, and sealing. Finally, panoramic scanning of fluorescent 20x samples was performed using TissueFAXS system. The data were analyzed by StrataQuest 7.1.1.129 analysis software, and the average optical density was analyzed by ImageJ software and analyzed statistically. Immunofluorescence staining used the following antibodies: Rabbit anti-human CD8(Abcam, ab101500, 1:100), rabbit anti-human Notch1(Abcam, ab51627, 1:150), rabbit anti-human GranzymeB (Abcam, ab255598, 1:3000), rabbit anti-human IFN-γ (HUABIO, ET1703-17, 1:100), the antibody required for immunohistochemical staining was DLL4(HUABIO, ER1706-29, 1:50).

### Statistical analysis

Flow results in this study were analyzed and processed using Flowjo software. All data were statistically analyzed using R software 4.2.2 and GraphPad Prism 8.0. Survival analysis was performed by drawing KM curves, and the survival difference between the two groups was assessed using the log-rank test. The chi-square test was used for categorical variables, while the t-test or non-parametric test was used for continuous variables based on whether they followed a normal distribution. ANOVA analysis was used for comparing multiple groups. p<0.05 indicates a statistically significant difference.

## Results

### CD8^+^T cell hub genes

The results of immune cell infiltration analysis revealed varying degrees of CD8^+^T cell infiltration among different HCC patients, with an overall higher proportion. In the correlation heatmap, we observed a positive correlation between CD8^+^T cells and activated memory CD4^+^T cells as well as follicular helper T cells, while a negative correlation was found with M0 macrophages and resting memory CD4^+^T cells (**Supplementary Figure 1**).

**Figure 2:**
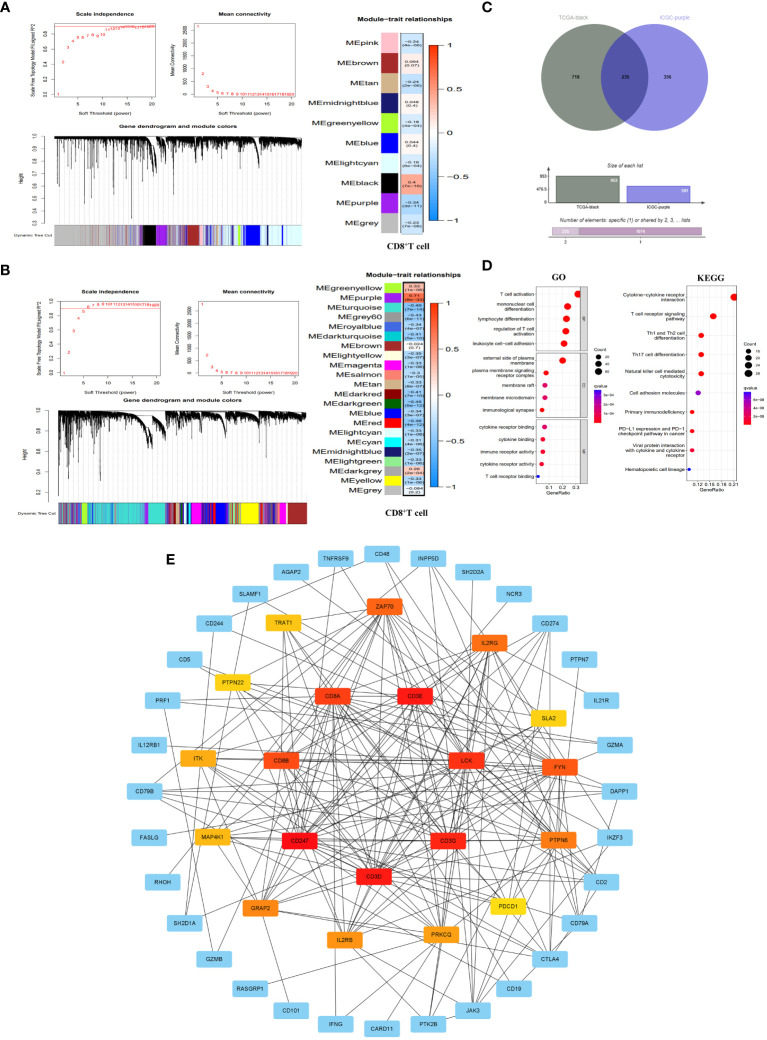
Identification of CD8^+^T cell hub genes and their molecular characteristics. **(A, B)** WGCNA results in the TCGA database **(A)** and ICGC database **(B)**. It included threshold selection, dynamic shear tree merging similar module genes and correlation analysis between module feature genes and CD8^+^T cells. **(C)** Venn diagram of CD8^+^T cell hub genes. **(D)** GO enrichment and KEGG pathway enrichment of the CD8^+^T cell hub genes. **(E)** PPI network construction of CD8^+^T cell hub gene. The PPI network was constructed by selecting the top 20 genes according to the MCC score. The redder the color, the more important the gene was.

The WGCNA analysis of the TCGA dataset revealed that a scale-free network distribution (R2 = 0.9) was observed when setting the power value to 14, indicating strong connectivity between genes. Subsequently, gene clustering and module merging resulted in the identification of 10 distinct gene populations. Notably, the black module exhibited a significant association with CD8^+^T cells (correlation 0.4, P=7e-16), encompassing a total of 953 genes (**Figure 2A**). Similarly, in the ICGC dataset, when the power value was set to 6, the connectivity between genes also followed a scale-free network distribution. This analysis resulted in the identification of 22 gene modules, with the purple module exhibiting the highest correlation with CD8^+^T cells (correlation 0.71, P=8e-33) and containing 591 genes (**Figure 2B**).

By integrating the WGCNA analysis outcomes from both TCGA and ICGC datasets, we identified a total of 235 genes (**Supplementary Table 2**) that were shared between the black module in TCGA and the purple module in ICGC. These genes serve as pivotal regulators of CD8^+^T cells, exerting significant regulatory influence on the function of CD8^+^T cells (**Figure 2C**). To further elucidate the molecular mechanisms underlying CD8^+^T cell hub genes, we conducted GO and KEGG enrichment analyses on these hub genes. The results of GO showed that the main biological processes (BP) involved T cell activation, monocyte differentiation, lymphocyte differentiation, regulatory T cell activation and leukocyte cell-cell adhesion. In terms of cellular component (CC), the enriched genes were predominantly located on the external side of the plasma membrane, plasma membrane signaling receptor complexe, membrane raft, membrane microdomain and immunological synapse. Regarding molecular functions (MF), they primarily exhibited immune receptor activity, cytokine binding and cytokine receptor binding and activity, as well as T cell receptor binding. KEGG pathway enrichment results showed that CD8^+^T hub genes were mainly enriched in primary immune deficiency, T cell receptor signaling pathway, cytokine-cytokine receptor interaction, NK cell-mediated cytotoxicity, Th1 and Th2 cell differentiation, Th17 cell differentiation, and tumor PD-L1 and PD-1 pathways (**Figure 2D**). At the same time, we have established a PPI network that highlights the top 20 genes in the MCC score (**Figure 2E**).

### Construction of a risk scoring model and nomogram

**Figure 3:**
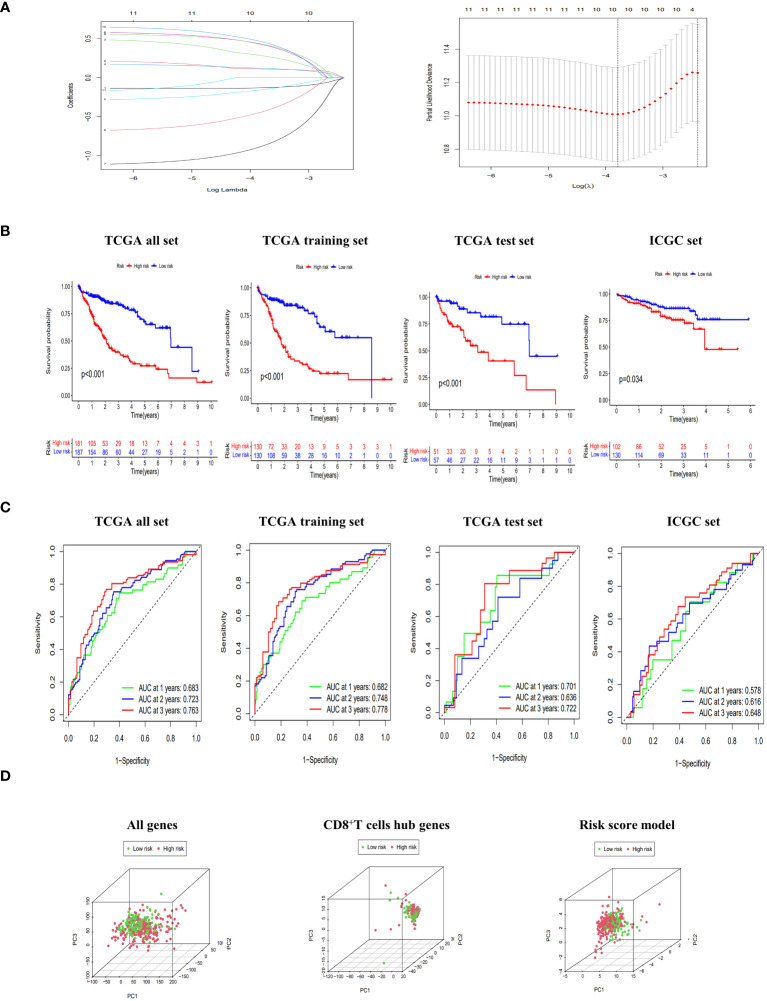
Construction and evaluation of risk scoring model. **(A)** Lasso regression to build risk scoring model. **(B)** The Kaplan-Meier (KM) curves of overall survival (OS) to compare the high-low risk groups in TCGA all set, TCGA training set, TCGA test set and ICGC set. **(C)** Receiver operating characteristic (ROC) curves of 1-, 2-, and 3-year OS to assess the discrimination ability of risk scoring model in TCGA all set, TCGA training set, TCGA test set and ICGC set. **(D)** principal component analysis (PCA) score plots of HCC patients in high-risk and low-risk groups to be divided according to all genes, CD8^+^T cell hub genes and risk score model.

As presented in **Supplementary Table 3**, the TCGA dataset was randomly partitioned into a training set (n=260) and a validation set (n=108), ensuring comparable baseline characteristics between the two datasets (p>0.05). Through lasso-cox regression, 8 genes were selected to form a risk scoring model (**Figure 3A**). The model formula was as follows: RiskScore=0.403*Exp (CCDC88C) +0.19*Exp (CD7)-0.311*Exp (FYN)+0.653*Exp (GATA3)-1.096*Exp (IL18RAP)-0.766*Exp (ITGB7) +0.575*Exp (MCOLN2) +0.648*Exp (TAF4B). The risk score model formula was employed to calculate the individual scores of each patient, and subsequently, the patients were categorized into different risk groups based on the median value of their scores. Additionally, external validation was conducted using the ICGC dataset. The KM curves all showed that the survival rate of the high-risk group was significantly lower than that of the low-risk group (p<0.05) (**Figure 3B**). Meanwhile, the ROC curve showed that the AUC values of the model at 1, 2 and 3 years were all high, which proved that the model had good discrimination ability (**Figure 3C**). In addition, PCA results showed that the risk score model better delineated the risk group of HCC patients compared to all genes and CD8^+^T cell hub genes (**Figure 3D**). Finally, the risk scoring model also showed significant discrimination among subgroups of age, sex, Grade and Stage (**Supplementary Figure 2**).

Based on the TCGA dataset, we constructed a nomogram by integrating risk scores with clinical indicators. Both univariate and multivariate Cox regression analyses demonstrated that the risk score significantly predicts patient prognosis. Furthermore, ROC curves revealed excellent discrimination and calibration abilities of the nomogram in our study (**Supplementary Figure 3**).

### Risk score subgroup clinical features and immune function

**Figure 4:**
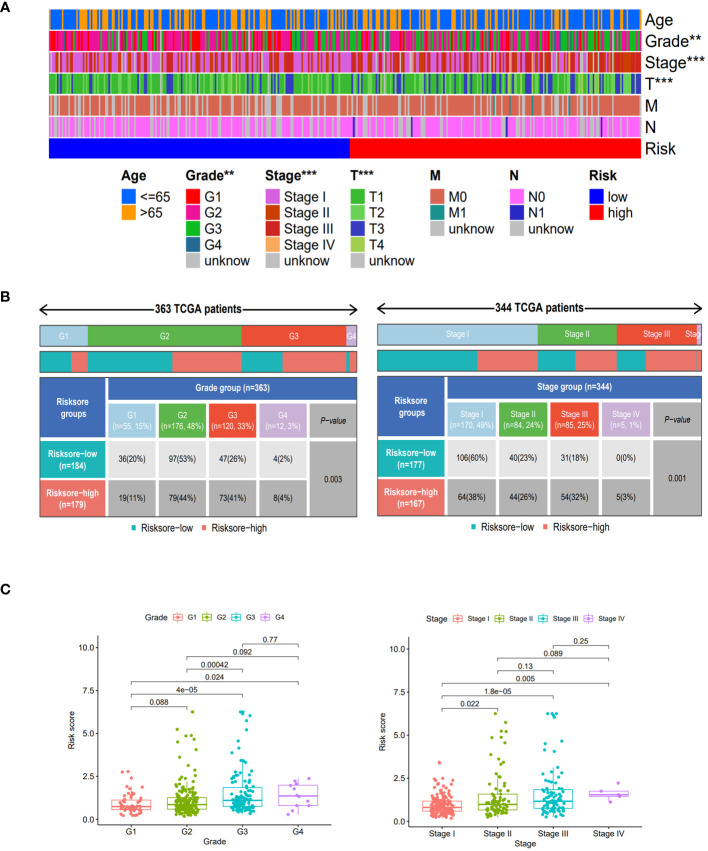
Clinical features of different risk sore subgroups. **(A)** Heatmap showing the clinical characteristics (age, grade, stage, T, M, N, etc.) and risk score groups of 368 HCC patients in TCGA set. **(B)** Heatmap and table showing the distribution of Grade subtypes (G1, G2, G3 and G4) and Stage subtypes (Stage I, Stage II, Stage III and Stage IV), between the risk score subgroups. **(C)** Comparison of different risk scores with Grade and Stage in the TCGA set. **, p < 0.01; ***, p<0.001.

Compared to the clinical characteristics of the high-low risk group in the risk scoring model, there were significant distribution differences observed between the two groups in terms of Grade, Stage, and T stage (p<0.05). Notably, the Stage I score was significantly lower than that of other stages, indicating that this risk score model could be well-suited for identifying early-stage HCC patients (**Figure 4**).

**Figure 5:**
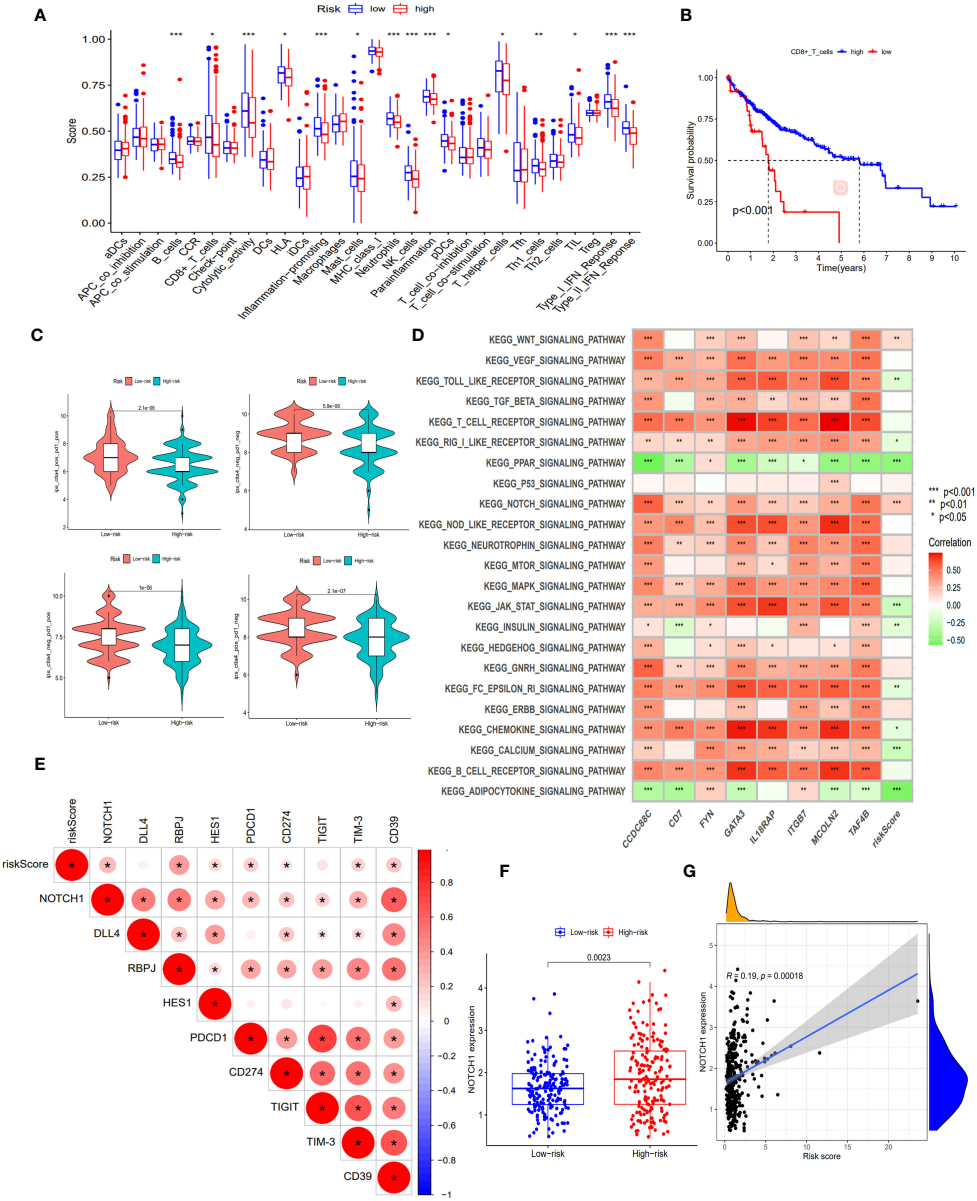
Exploration of immune infiltration characteristics and exhaustion pathways in risk score subgroups. **(A)** Proportion of immune cell infiltration in different risk score subgroups, scatter represents the score, the middle horizontal line of the box diagram represents the median, and the upper and lower horizontal lines represent 25% and 75%. **(B)** The KM curve compared the survival rate of CD8^+^T cell subgroups. **(C)** Differences in immunotherapy (anti-PD-1 and anti-CTLA-4) between high and low risk score subgroups. **(D)** Heatmap of GSVA enrichment results. The darker the color, the more significant the enrichment, red represents a positive correlation, green represents a negative correlation. **(E)** Correlation between Notch pathway and exhaustion genes and risk scores. The darker the color, the more significant the enrichment, the redder the higher the degree of positive correlation. **(F)** Expression of NOTCH1 in risk score subgroups. **(G)** Correlation of NOTCH1 with risk scores. *, p < 0.05; **, p < 0.01; ***, p<0.001.

When comparing the immune function between the two groups, our findings revealed that the low-risk group demonstrated significantly elevated levels of multiple immune functions in comparison to the high-risk group. These immune functions included B cells, CD8^+^T cells, Cytolytic activity, HLA, pro-inflammatory, mast cells, Neutrophils, NK cells, parainflammation, pDCs, helper T cells, Th1 cells, TIL, type I interferon response and type II interferon response (**Figure 5A**); By stratifying the CD8^+^T cell levels into high and low categories, we observed a significantly elevated survival rate among patients in the high-level CD8^+^T cell group compared to those in the low-level group (**Figure 5B**); Patients in the low-risk cohort demonstrated improved outcomes following treatment with anti-PD-1, anti-CTLA-4, and other immunotherapies (**Figure 5C**); The enrichment analysis of GSVA pathway revealed a significant positive correlation between risk scores and the expression of the Notch signaling pathway and Wnt signaling pathway (**Figure 5D**), indicating their high expression in the high-risk groups.

### Exhaustion regulation of Notch signaling pathway

In this study, we observed an inhibition of immune function in the high-risk group and identified a significant enrichment of the Notch signaling pathway within this group. To gain deeper insights into the impact of this pathway on immune function, we investigated the correlation between genes involved in the Notch signaling pathway (Notch1, DLL4, RBPJ, and HES1) and both risk scores as well as known exhaustion pathway genes (PDCD1, CD274, TIGIT, TIM-3, and CD39). The correlation analysis revealed significant positive associations between risk scores and Notch1, RBPJ, HES1, PDCD1, CD274, TIM-3, and CD39 expression levels (**Figure 5E**), indicating a positive relationship between the Notch pathway and exhaustion genes. Furthermore, Notch1 expression was significantly higher in the high-risk group compared to the low-risk group (p<0.05), with risk score showing a significant positive correlation with Notch1 expression (R=0.19, p<0.05) (**Figures 5F, G**). These findings suggest that the involvement of the Notch signaling pathway in exhaustion regulation is evident and highlight the potential utility of Notch1-positive cells as markers for identifying high-risk exhaustion cells.

### Relationship between Notch1 and CD8^+^T exhaustion

**Figure 6:**
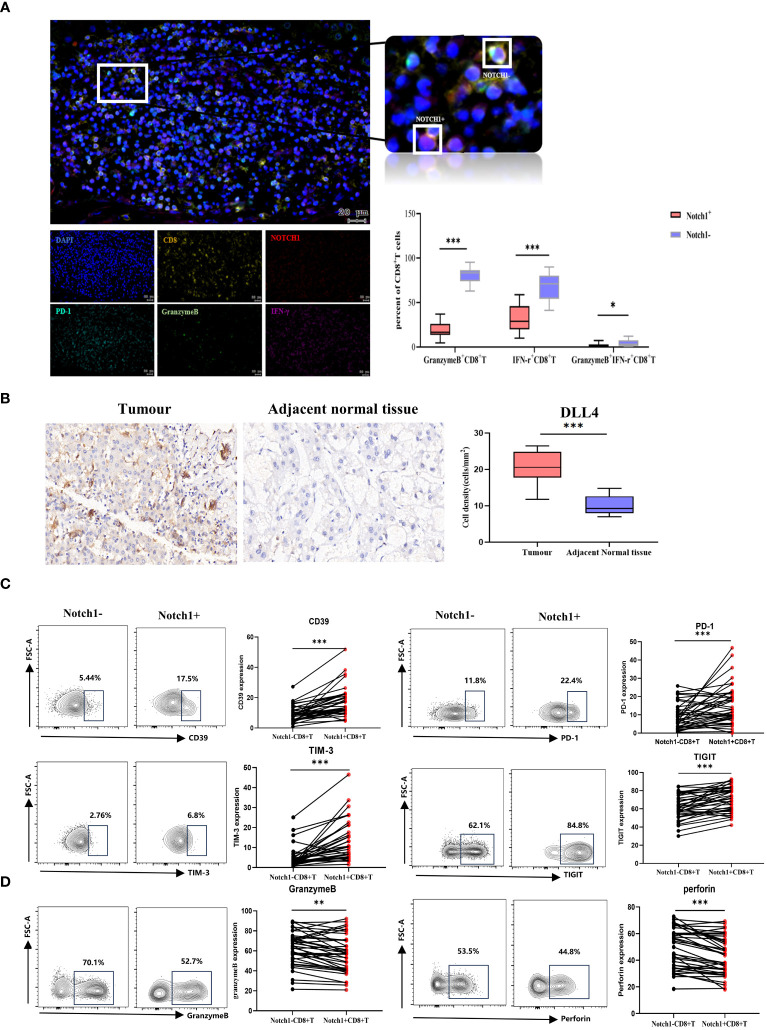
Clinical samples verified differences in phenotype and function between Notch1^+^CD8^+^T cells and Notch1^-^CD8^+^T cells. **(A)** Immunofluorescence and statistical graph of HCC tissues. **(B)** Immunohistochemical and statistical graph of DLL4 in tumor and paratumor tissues. The exhaustion phenotype **(C)** and function **(D)** of CD8^+^T cells in peripheral blood of HCC patients were analyzed by flow cytometry. *, p < 0.05; **, p < 0.01; ***, p<0.001.

We evaluated the phenotype and functional characteristics of CD8^+^T cells in HCC patients by immunofluorescence, immunohistochemistry, and flow cytometry. The immunofluorescence results demonstrated a significant decrease in the levels of GranzymeB and IFN-γ expression in Notch1^+^CD8^+^T cells compared to Notch1^-^CD8^+^T cells (p<0.05) (**Figure 6A**), indicating that Notch1-positive cells exhibited diminished functionality relative to their Notch1-negative counterparts. Furthermore, the percentage of Notch1^+^CD8^+^T cells and PD-1^+^CD8^+^T cells within tumor tissues exhibited a greater magnitude than that observed in adjacent normal tissues (**Supplementary Figure 4**). Based on immunohistochemical analysis, DLL4, a ligand of the Notch signaling pathway, exhibited significant upregulation in cancer tissues compared to adjacent tissues(p<0.05) (**Figure 6B**). Additionally, flow cytometry results indicated that Notch1^+^CD8^+^T cells exhibited significantly higher expressions of CD39, PD-1, TIM-3, and TIGIT compared to Notch1^-^CD8^+^T cells, while the expressions of GranzymeB and Perforin were significantly decreased (p<0.05) (**Figures 6C, D**), indicating that cells positive for Notch1 in peripheral blood also had low function.

**Figure 7:**
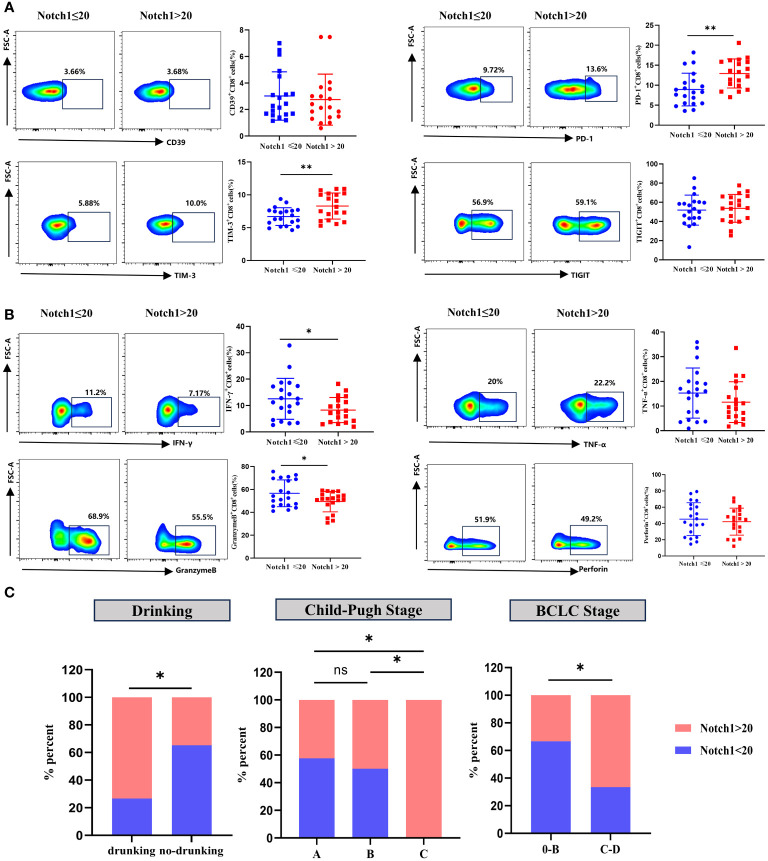
Clinical samples verified the phenotype and functional changes of CD8^+^T cells in Notch1 high and low subgroups. The exhaustion phenotype **(A)** and function **(B)** of CD8^+^T cells in peripheral blood of HCC patients were analyzed by flow cytometry. *, p < 0.05; **, p < 0.01. **(C)** Differences in Drinking, Child-Pugh Stage, and BCLC Stage between high and low Notch1 subgroups. *, p < 0.05; **, p < 0.01.

Clinical HCC patients were divided into two groups, high and low, based on the median value of Notch1 levels. The phenotype and function of CD8^+^T cells were compared between these two groups. The findings revealed that the levels of PD-1 and TIM-3 in CD8^+^T cells were significantly higher in the high-level group compared to the low-level group (**Figure 7A**). In contrast, the expression of IFN-γ and GranzymeB was significantly decreased (**Figure 7B**). Moreover, a comparative analysis of the clinical characteristics was conducted between the high and low groups. The results demonstrated that patients with high levels of Notch1 had a higher alcohol consumption rate and were predominantly advanced HCC patients with Child-Pugh C and BCLC C-D stage (**Figure 7C**; **Supplementary Table 4**). This indicates a greater exhaustion of CD8^+^T cells in the cohort with a high percentage of Notch1. Moreover, most patients in this group had a history of alcohol consumption and were mostly in the advanced stage of the disease.

## Discussion

Early detection and treatment play a crucial role in improving the survival rate of HCC patients. In recent years, significant advancements have been made in precision medicine, particularly with the widespread use of gene information technology. By analyzing the gene transcriptome, tumors can be identified at an early stage, enabling effective diagnosis and treatment, ultimately leading to a substantial improvement in patient survival rates (17). The TME, which encompasses various cell types including immune cells, has a profound impact on tumor outcomes and prognosis (18). During tumor development, lymphocytes migrate from the blood and infiltrate the tumor growth area, actively participating in tumor immunity (19, 20). Among these lymphocytes, CD8^+^T cells serve as the primary anti-tumor effector cells, capable of directly eliminating tumor cells through GranzymeB and Perforin, as well as indirectly influencing tumor cells by secreting cytokines (21). However, in the anti-tumor process, CD8^+^T cells experience long-term chronic inflammation stimulation, resulting in high expression of co-inhibitory molecules and a suppressed cell function, leading to tumor immune escape (22), which ultimately impacts patient survival. Therefore, understanding the regulation mechanism of CD8^+^T cell exhaustion holds significant importance.

In this study, we have successfully identified 235 hub genes of CD8^+^T cells through a comprehensive analysis of immune cell infiltration. The GO and KEGG enrichment analyses have revealed that these hub genes play crucial roles in the activation and differentiation processes of immune cells, as well as in the cytokine activation pathways. Furthermore, our findings highlight their significant involvement in regulating the expression of PD-L1 and PD-1 signaling pathways within TME.

Eight genes (CCDC88C, CD7, FYN, GATA3, IL18RAP, ITGB7, MCOLN2 and TAF4B) were screened by lasso-cox, most of which were closely related to the occurrence and development of tumors (23–26). Additionally, they play a crucial role in the development and differentiation of T cells (27–30). Based on the analysis of these eight genes, we developed a risk score model that effectively stratifies the HCC population into high and low-risk groups using the median risk score as a threshold. The two groups showed significant differences in survival time, and the model demonstrated excellent discrimination ability as indicated by the ROC value and PCA analysis. Furthermore, when comparing clinical characteristics between the two groups, we observed that patients in the high-risk group exhibited more aggressive tumor malignancy. Notably, the risk score demonstrated exceptional efficacy in distinguishing early-stage and advanced-stage patients, thereby enhancing its potential as a valuable prognostic indicator. Furthermore, the risk score model displayed strong applicability across different age groups, genders, and tumor stages. These findings suggested that the risk score model has broad clinical relevance and remarkable discrimination ability. Additionally, the comparison of immune cell infiltration between the two groups revealed that the high-risk group exhibited lower immune function, reduced efficacy of immunotherapy, increased expression of the Notch signaling pathway, and a significant positive correlation with exhaustion-related genes. Therefore, it is plausible to speculate that the activation of the Notch pathway may lead to immune function suppression in patients, resulting in an exhausted immune microenvironment. This exhausted immune microenvironment could potentially be the underlying cause of the low survival rate observed in the high-risk group.

Notch signaling pathway is a highly conserved pathway that involves 5 ligands (Jagged1, Jagged2, Delta-like1 (DLL1), Delta-like3 (DLL3), and Delta-like4 (DLL4)) and 4 receptors (Notch1-4). The pathway also encompasses intracellular effector molecules and downstream hub genes that are pivotal for embryonic development and tissue homeostasis. It plays a significant role in regulating various cell types, including immune cells, while being involved in the processes of cell proliferation and differentiation (31, 32). Numerous studies have demonstrated the correlation between the aberration of the Notch signaling pathway and the onset and progression of cancer. Importantly, it should be noted that this pathway exhibits dual roles in tumor development by acting both as a promoter or an inhibitor depending on specific conditions (33). Previous investigations have highlighted the crucial involvement of the Notch pathway in diverse tumor types such as medulloblastoma (34) and T-cell leukemia (35). Additionally, the Notch pathway is also involved in T cell development. During the initial stages of T cell development, the activation of Notch signaling prompts lymphoid progenitor cells to differentiate into the T cell lineage rather than the B cell lineage (36). Research studies have demonstrated that within a specific thymus microenvironment, the consistent activation of Notch signaling through the expression of DLL4 is crucial for initiating T cell development (37). This signaling pathway suppresses the differentiation of T lymph progenitor cells into γδT cells, while promoting the development of αβT cells (38). The pivotal involvement of Notch signaling in the intricate processes of T cell development and differentiation has been firmly established. Moreover, the Notch signaling pathway exerts a profound regulatory influence on the maturation of CD8^+^T cells. Research has demonstrated that the Notch1 signal can hinder the development of DP thymocytes into CD4^+^CD8^-^T cells while facilitating the development of CD4^-^CD8^+^T cells, and this is primarily attributed to the alteration of T cell receptor (TCR) signal expression in DP thymocytes by the Notch signal, thereby promoting this biological process (39, 40). Notch signaling also controls the generation and differentiation of efficient CD8^+^T cells (41), and plays an important role in the exhaustion of CD8^+^T cells. The regulation of Notch signaling pathway exhaustion has been reported in a variety of tumors, and previous studies have shown that Notch1 knockout plays an important role in promoting the formation of esophageal squamous cell carcinoma and immune escape (42). When Notch signaling is inhibited, the expression of PD-1 on the surface of CD8^+^T cells is also inhibited (43), indicating that Notch signaling pathway has potential role in controlling the expression of PD-1 and reversing the function of CD8^+^T cells. In conclusion, Notch signaling has a bidirectional regulatory effect on the development of CD8^+^T cells, which can both induce the development of CD8^+^T cells at an early stage and inhibit the function of CD8^+^T cells under certain conditions.

In this study, we observed a significant enrichment of the Notch pathway in the high-risk group, which exhibited a strong positive correlation with the risk score and expression of exhaustion molecules. Our clinical sample analysis revealed that the expression of Notch pathway receptor Notch1 and ligand DLL4 was significantly higher in HCC tissues compared to para-cancer tissues. Flow cytometry results also demonstrated a higher expression of co-inhibitory molecules (CD39, TIM-3, PD-1, and TIGIT) on Notch1^+^CD8^+^T cells in peripheral blood compared to Notch1^-^CD8^+^T cells. Additionally, we compared the phenotype and functional expression of CD8^+^T cells between the high-Notch1 and low-Notch1 groups. We observed a positive correlation between elevated levels of Notch1 expression and increased expression of co-inhibitory molecules on the cell surface, as well as a concurrent decrease in cellular function. These findings suggest that activation of the Notch pathway can induce an upregulation of co-inhibitory molecule expression on CD8^+^T cells, ultimately leading to impaired functionality. Importantly, these results are consistent with those obtained from publicly available databases, further supporting their validity and significance within the scientific community. The identification of novel immune targets represents a crucial approach to addressing the limitations of current immunotherapy efficacy and resistance to ICIs. The advancement in single cell sequencing technology has facilitated this process. Prior research has demonstrated that utilizing genome and transcriptome sequencing as a guide can assist patients in receiving personalized targeted therapy, leading to significant enhancements in treatment efficiency (44). This study has constructed a risk score model utilizing eight genes linked to tumor progression and T cell differentiation. Clinically, the detection of their expression can effectively predict the prognosis of patients with HCC, reflecting immune strength and tumor progression to some extent, and guiding personalized treatment strategies. Furthermore, our study has identified Notch1 expression as a potential biomarker for CD8^+^T cell exhaustion, suggesting that Notch1 may serve as a promising immune target for the development of molecularly targeted therapeutics in the future. When combined with other ICIs, it is anticipated to enhance the overall efficacy of immunotherapy in patients with HCC.

Furthermore, our investigation proposes that alcohol consumption may contribute to aberrant activation of the Notch pathway. Alcohol intake is closely linked with hepatic injury (45), and the age-standardized mortality rate for alcoholic HCC has been progressively increasing. It is estimated that 19% of global HCC deaths in 2019 were attributable to alcohol (46). Alcohol has been found to induce angiogenesis by stimulating the conduction of the Notch signaling pathway (47). Tumor angiogenesis represents a crucial step in tumor progression, as tumors remain quiescent for an extended period before initiating vascularization (48).

There are two limitations associated with this study. Firstly, it is important to acknowledge that the scope of this investigation was limited to HCC patients, and further research is required to determine whether these findings can be equally advantageous for other tumor types. Secondly, while the study primarily focused on gene regulation of CD8^+^T cells, it did not extensively explore additional immune cell analysis. Therefore, future investigations should aim to elucidate the prognostic significance of other immune cell populations.

## Conclusion

In this study, we investigated the prognostic value of CD8^+^T cells in patients with HCC. We developed a prognostic scoring system that accurately predicts the survival rate of HCC patients and distinguishes between early, intermediate, and advanced-stage patients. Surprisingly, our risk scoring system also identifies immunodepleted HCC groups and, to some extent, helps identify immunotherapy advantaged groups Furthermore, our results suggested that the Notch pathway may be the main regulatory mechanism involved in the exhaustion of CD8^+^T cells. These findings provide valuable insights for future immunotherapy targeting the Notch pathway.

## Data availability statement

The original contributions presented in the study are included in the article/**Supplementary Material**, further inquiries can be directed to the corresponding authors.

## Ethics statement

The studies involving humans were approved by the Ethics Committee of Beijing Ditan Hospital Affiliated to Capital Medical University. The studies were conducted in accordance with the local legislation and institutional requirements. The participants provided their written informed consent to participate in this study.

## Author contributions

QP: Conceptualization, Data curation, Formal analysis, Funding acquisition, Investigation, Methodology, Project administration, Resources, Software, Supervision, Validation, Visualization, Writing – original draft, Writing – review & editing. LY: Conceptualization, Data curation, Formal analysis, Funding acquisition, Investigation, Methodology, Project administration, Resources, Software, Supervision, Validation, Visualization, Writing – original draft, Writing – review & editing. XL: Writing – review & editing. HY: Data curation, Formal analysis, Methodology, Writing – review & editing. YX: Investigation, Software, Validation, Writing – review & editing. XC: Data curation, Investigation, Methodology, Writing – review & editing. YW: Investigation, Methodology, Software, Writing – review & editing. JD: Project administration, Supervision, Writing – review & editing, Writing – original draft. ZY: Project administration, Supervision, Visualization, Writing – review & editing, Writing – original draft.
