# Supplementary material for: Prognostic value of CD8+T cells related genes and exhaustion regulation of Notch signaling pathway in hepatocellular carcinoma
Source: Front Immunol. 2024 Apr 8;15:1375864. doi: 10.3389/fimmu.2024.1375864 (PMC11033358; doi:10.3389/fimmu.2024.1375864)
Supplement: Supplementary file 1 [file DataSheet_1.docx]

## Supplementary Tables

### Table S1 Clinical characteristics of the TCGA and ICGC cohorts.

| **Variable** | **Type** | **TCGA** | **ICGC** |
| --- | --- | --- | --- |
|  |  | **(n=368)** | **(n=232)** |
| Age | <=65 | 231(62.77%) | 90(38.79%) |
|  | >65 | 137(37.23%) | 142(61.21%) |
| Gender | FEMALE | 121(32.88%) |  |
|  | MALE | 247(67.12%) |  |
| Grade | G1 | 55(14.95%) |  |
|  | G2 | 176(47.83%) |  |
|  | G3 | 120(32.61%) |  |
|  | G4 | 12(3.26%) |  |
|  | unknow | 5(1.36%) |  |
| Stage | Stage I | 170(46.2%) | 36(15.52%) |
|  | Stage II | 84(22.83%) | 106(45.69%) |
|  | Stage III | 85(23.1%) | 71(30.6%) |
|  | Stage IV | 5(1.36%) | 2(8.19%) |
|  | unknow | 24(6.52%) |  |
| T | T1 | 180(48.91%) |  |
|  | T2 | 92(25%) |  |
|  | T3 | 80(21.74%) |  |
|  | T4 | 13(3.53%) |  |
|  | unknow | 3(0.82%) |  |
| M | M0 | 264(71.74%) |  |
|  | M1 | 4(1.09%) |  |
|  | unknow | 100(27.17%) |  |
| N | N0 | 250(67.93%) |  |
|  | N1 | 4(1.09%) |  |
|  | unknow | 114(30.98%) |  |

### Table S2 235 CD8+T cell associated genes.

| ZC3H12D | C11orf21 | CD3G | CTLA4 | FGD3 | HAPLN3 |
| --- | --- | --- | --- | --- | --- |
| ZNF683 | C16orf54 | CD40LG | CTSW | FMNL1 | HLA-DOB |
| ACAP1 | CARD11 | CD48 | CXCL10 | FYN | HLA-F |
| AGAP2 | CCDC88B | CD5 | CXCL11 | GATA3 | HSH2D |
| AIM2 | CCDC88C | CD52 | CXCL13 | GBP1 | ICOS |
| AKAP5 | CCL5 | CD6 | CXCL9 | GBP4 | IDO1 |
| AKNA | CCND2 | CD69 | CXCR3 | GBP5 | IFNG |
| ANO9 | CCR2 | CD7 | CXCR6 | GNLY | IKZF1 |
| APOBEC3G | CCR5 | CD70 | CYTIP | GP1BA | IKZF3 |
| APOBEC3H | CCR7 | CD79A | DAPP1 | GPR132 | IL12RB1 |
| ARHGAP25 | CD101 | CD79B | DEF6 | GPR171 | IL16 |
| ARHGAP9 | CD19 | CD8A | DGKA | GPR174 | IL18BP |
| ASB2 | CD2 | CD8B | DOCK8 | GPR18 | IL18RAP |
| ATP2A3 | CD244 | CD96 | DUSP2 | GPR68 | IL21R |
| BATF2 | CD247 | CHI3L2 | EMB | GRAP2 | IL2RB |
| BCL11B | CD27 | CLEC2D | EOMES | GZMA | IL2RG |
| BIN2 | CD274 | CORO1A | ETV7 | GZMB | INPP5D |
| BTN3A1 | CD38 | CPNE5 | FASLG | GZMH | IRF1 |
| BTN3A2 | CD3D | CRTAM | FCHO1 | GZMK | IRF4 |
| BTN3A3 | CD3E | CST7 | FCRL6 | GZMM | ISG20 |
| ITGAL | MAL | PIK3CD | RASGRP1 | SLC38A5 | TNFRSF9 |
| ITGB7 | MAP4K1 | PIM2 | RHOH | SP140 | TOX |
| ITK | MCOLN2 | PLA2G2D | RIPK3 | SPN | TRAF3IP3 |
| ITPRIPL1 | MEI1 | POU2AF1 | RNF166 | SPOCK2 | TRAT1 |
| JAK3 | MMP25 | PPP1R16B | RUNX3 | STAT4 | TRERF1 |
| JSRP1 | MS4A1 | PRDM1 | S1PR4 | STK17B | TRIM69 |
| KCNJ10 | MYO1G | PRF1 | SASH3 | SYTL1 | TSPAN32 |
| KCNN4 | MZB1 | PRKCH | SCML4 | SYTL3 | UBASH3A |
| KLRF1 | NCR3 | PRKCQ | SELL | TAF4B | UNC13D |
| KLRG1 | NELL2 | PSMB10 | SEMA4A | TAGAP | VILL |
| LAG3 | NKG7 | PSMB9 | SEMA4D | TAP1 | WDFY4 |
| LAX1 | NLRC3 | PSTPIP1 | SH2D1A | TBC1D10C | XCL2 |
| LCK | NLRC5 | PTK2B | SH2D2A | TBX21 | ZAP70 |
| LGALS2 | P2RX1 | PTPN22 | SIDT1 | THEMIS | ZBED2 |
| LIMD2 | P2RX5 | PTPN6 | SIRPG | TIGIT | ZBP1 |
| LPXN | P2RY10 | PTPN7 | SIT1 | TMC6 | LY75 |
| LRRK1 | PARP15 | PYHIN1 | SLA2 | TMC8 | PDCD1 |
| LTA | PATL2 | RAB33A | SLAMF1 | TNFRSF13C | RASAL3 |
| LTB | PBX4 | RAC2 | SLAMF6 | TNFRSF17 | SLAMF7 |
| TNFRSF18 |  |  |  |  |  |

### Table S3 TCGA dataset modeling baseline characteristics.

| **Variable** | **Type** | **Total set** | **Validation set** | **Training set** | **Pvalue** |
| --- | --- | --- | --- | --- | --- |
|  |  | **(n=368)** | **(n=108)** | **(n=260)** |  |
| Age | <=65 | 231(62.77%) | 70(64.81%) | 161(61.92%) | 0.6861 |
|  | >65 | 137(37.23%) | 38(35.19%) | 99(38.08%) |  |
| Gender | FEMALE | 121(32.88%) | 42(38.89%) | 79(30.38%) | 0.1444 |
|  | MALE | 247(67.12%) | 66(61.11%) | 181(69.62%) |  |
| Grade | G1 | 55(14.95%) | 23(21.3%) | 32(12.31%) | 0.141 |
|  | G2 | 176(47.83%) | 45(41.67%) | 131(50.38%) |  |
|  | G3 | 120(32.61%) | 35(32.41%) | 85(32.69%) |  |
|  | G4 | 12(3.26%) | 3(2.78%) | 9(3.46%) |  |
|  | unknow | 5(1.36%) | 2(1.85%) | 3(1.15%) |  |
| Stage | Stage I | 170(46.2%) | 47(43.52%) | 123(47.31%) | 0.8597 |
|  | Stage II | 84(22.83%) | 25(23.15%) | 59(22.69%) |  |
|  | Stage III | 85(23.1%) | 27(25%) | 58(22.31%) |  |
|  | Stage IV | 5(1.36%) | 2(1.85%) | 3(1.15%) |  |
|  | unknow | 24(6.52%) | 7(6.48%) | 17(6.54%) |  |
| T | T1 | 180(48.91%) | 50(46.3%) | 130(50%) | 0.4453 |
|  | T2 | 92(25%) | 27(25%) | 65(25%) |  |
|  | T3 | 80(21.74%) | 28(25.93%) | 52(20%) |  |
|  | T4 | 13(3.53%) | 2(1.85%) | 11(4.23%) |  |
|  | unknow | 3(0.82%) | 1(0.93%) | 2(0.77%) |  |
| M | M0 | 264(71.74%) | 77(71.3%) | 187(71.92%) | 1 |
|  | M1 | 4(1.09%) | 1(0.93%) | 3(1.15%) |  |
|  | unknow | 100(27.17%) | 30(27.78%) | 70(26.92%) |  |
| N | N0 | 250(67.93%) | 71(65.74%) | 179(68.85%) | 0.1388 |
|  | N1 | 4(1.09%) | 3(2.78%) | 1(0.38%) |  |
|  | unknow | 114(30.98%) | 34(31.48%) | 80(30.77%) |  |

### Table S4 Differences in clinical features between high and low Notch1 subgroups.

| **Variables** | **Notch1≤20 (n=20)** | **Notch1>20 (n=19)** | **P value** |
| --- | --- | --- | --- |
| Sex(male/female) | 14/6 | 16/3 | 0.292 |
| Age (≤60/>60year) | 8/12 | 9/10 | 0.643 |
| Smoking (yes/no) | 4/16 | 8/11 | 0.135 |
| Drinking (yes/no) | 4/16 | 11/8 | 0.015* |
| Hypertension (yes/no) | 12/8 | 8/11 | 0.264 |
| Heart disease (yes/no) | 0/20 | 2/17 | 0.136 |
| Diabetes (yes/no) | 8/12 | 4/15 | 0.200 |
| UGIB (yes/no) | 1/19 | 1/18 | 0.970 |
| Portal hypertension (yes/no) | 4/16 | 8/11 | 0.135 |
| WBC (≤4×109/>4×109/L) | 11/9 | 8/11 | 0.421 |
| NLR (≤2.41/>2.41) | 11/9 | 6/13 | 0.140 |
| ALP (≤104/>104 U/L) | 15/5 | 9/10 | 0.076 |
| PLT (≤100×109/>100×109/L) | 11/9 | 11/8 | 0.855 |
| ALT (≤50/＞50 U/L) | 18/2 | 16/3 | 0.589 |
| AST (≤40/>40 U/L) | 17/3 | 13/6 | 0.219 |
| INR (≤1.2/>1.2) | 9/11 | 7/12 | 0.605 |
| AFP (≤400/>400 ng/ml) | 16/4 | 16/3 | 0.732 |
| CRP (≤5/>5 mg/L) | 17/3 | 13/6 | 0.219 |
| γ-GGT (≤60/>60 U/L) | 16/4 | 10/9 | 0.07 |
| Child-Pugh Stage (A/B/C) | 15/5/0 | 11/5/3 | 0.166 |
| BCLC Stage (0-B/C-D) | 14/6 | 7/12 | 0.038* |

**Note**: *, p<0.05.

## Supplementary Figure


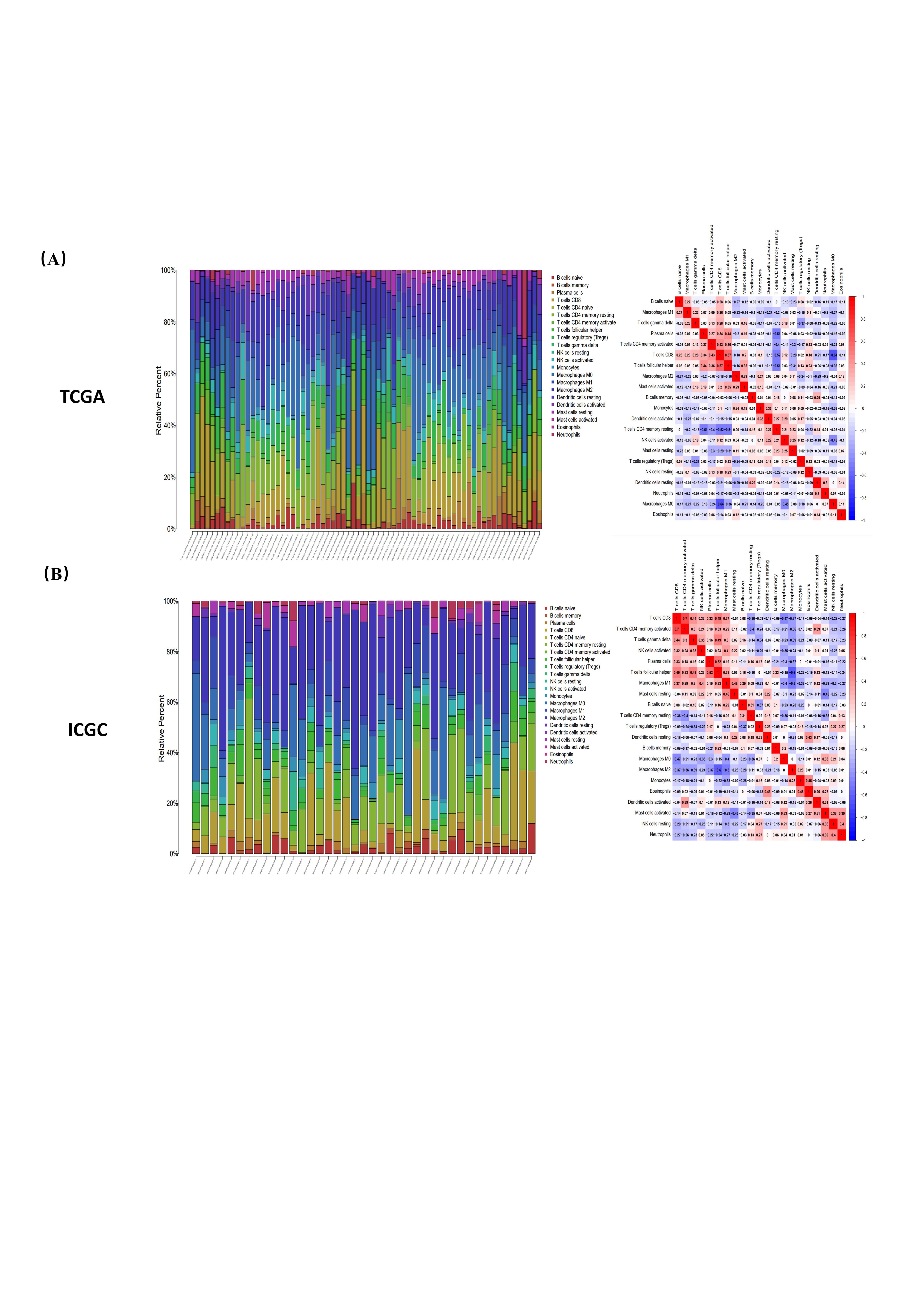


### Figure S1. Analysis of immune cell infiltration in HCC patients.

Histogram and correlation heat map of immune cell proportion in TCGA dataset (A) and ICGC dataset (B). In the bar chart, the horizontal axis represents each sample, while the vertical axis denotes the composition fraction of 22 distinct immune cell types. The color scheme is employed to differentiate between these cell types. In the correlation heat map, red signifies a positive correlation, blue indicates a negative correlation, and the intensity of color reflects the strength of this association.


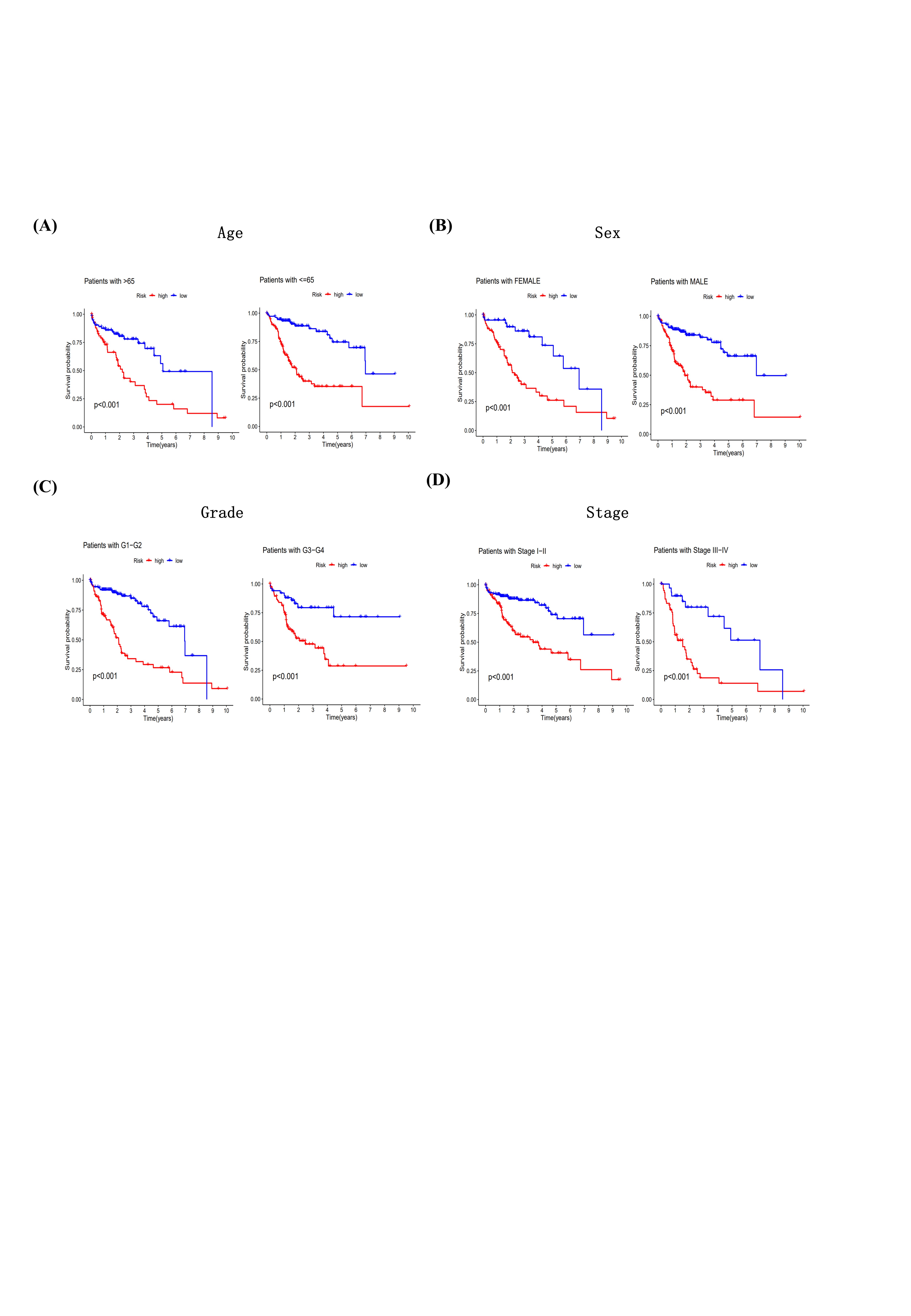


### Figure S2. Applicability of the high and low risk groups to each clinical feature subtype.

The Kaplan-Meier (KM) curves for age (A)、sex(B)、grading (C) and staging (D) Cancer. The vertical axis represents the survival rate, while the horizontal axis denotes the survival time.


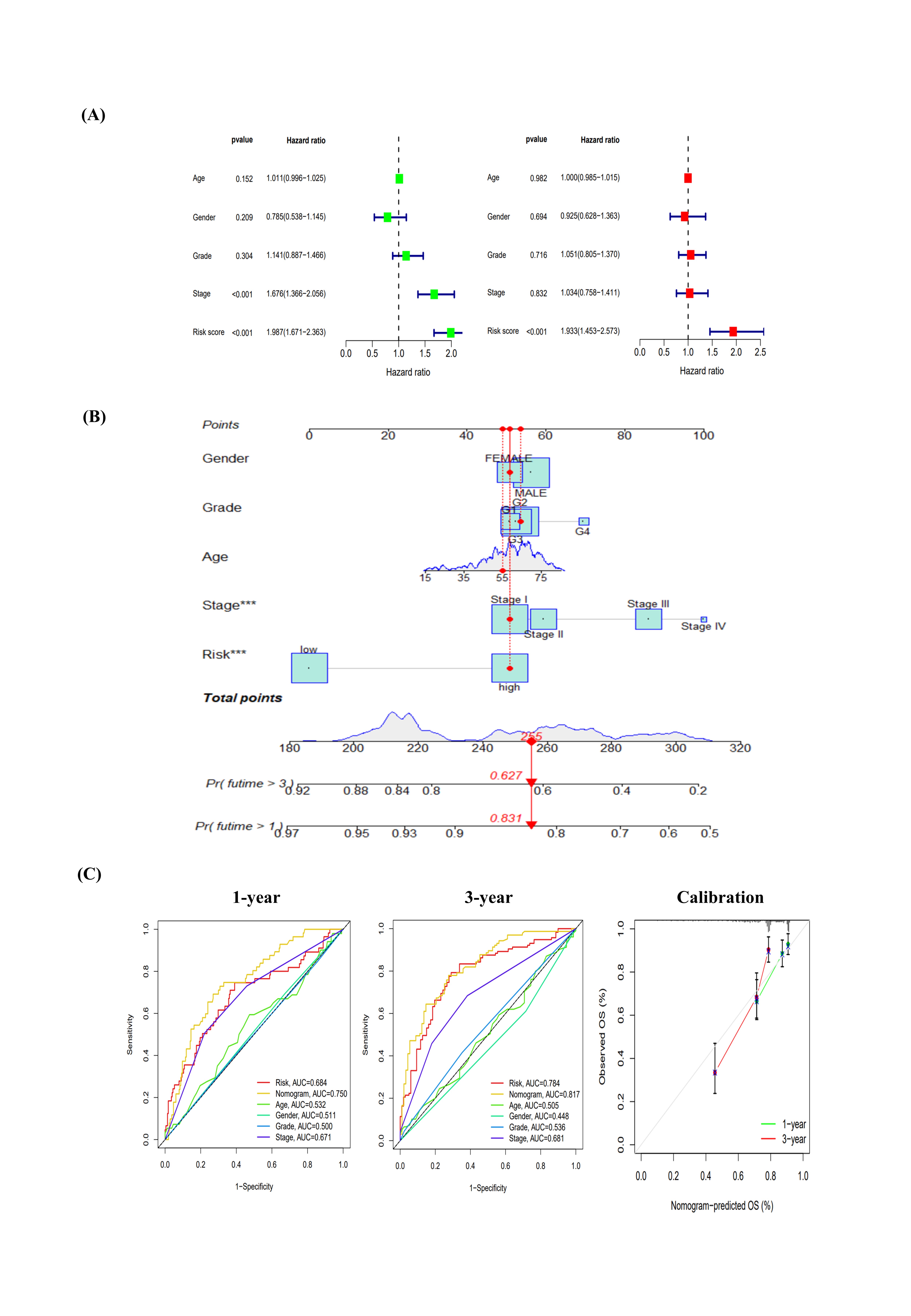


### Figure S3. Construction and evaluation of nomogram.

(A) Unifactor forest plot (left) and multifactor forest plot (right). HRs above one indicates that a variable is negatively with survival time; (B)Nomogram. Based on the actual patient's indicators, the corresponding index scores were determined and aggregated to derive the cumulative score along the vertical axis. The predicted survival rate is then identified at the intersection point of this vertical line with the survival axis; (C) Receiver operating characteristic (ROC) curves and calibration curves to assess the predictive capacity of the nomogram in estimating the survival rates at 1-year and 3-year. ***, p<0.001.


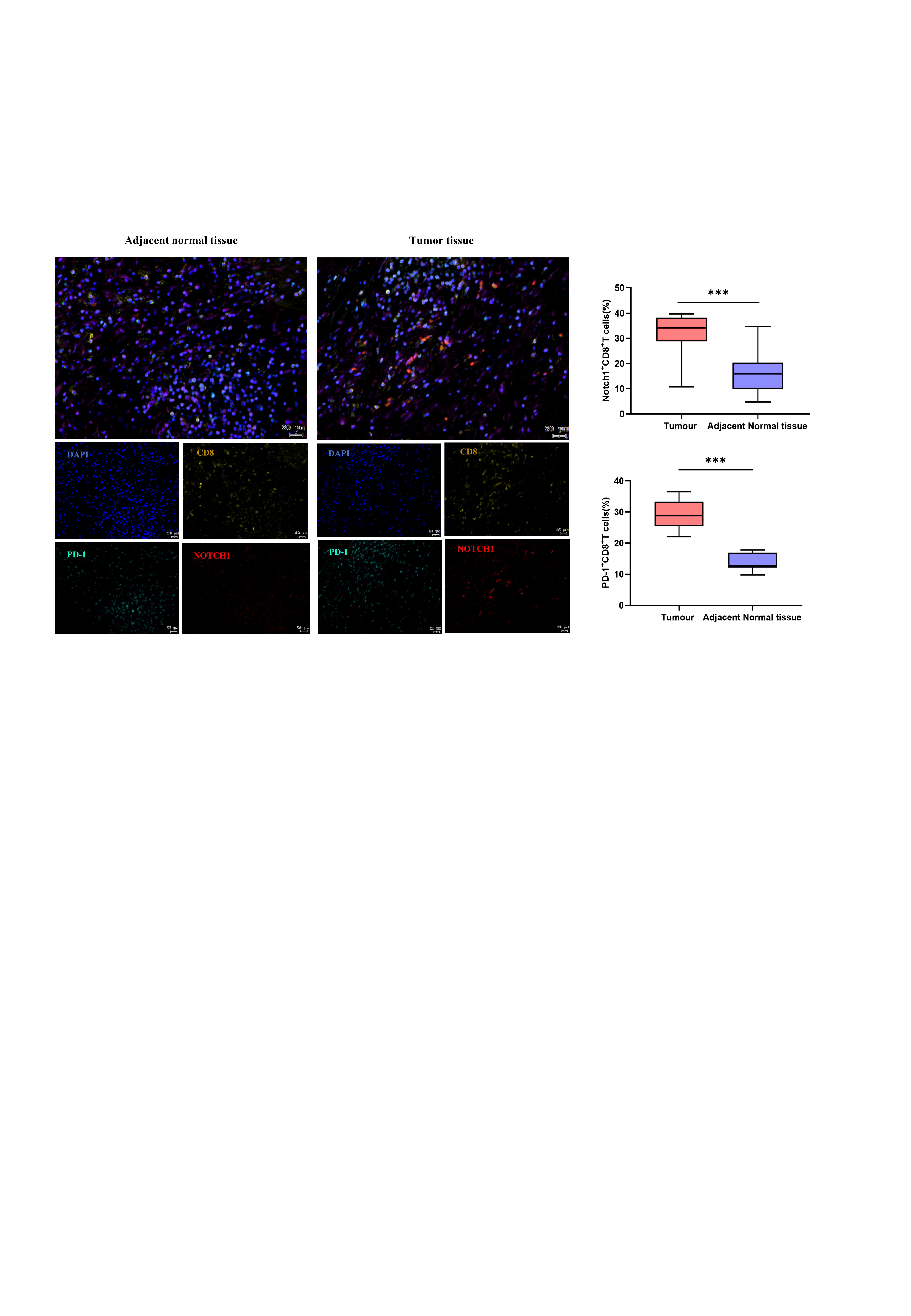


### Figure S4. Differences in expression of Notch1 and PD-1 on CD8+T cells in tumour and adjacent normal tissue.

(A) Multicolor immunofluorescence diagram of Notch1^+^CD8^+^T cells and PD-1^+^CD8^+^T cells; (B) Statistical analysis charts. ***, p<0.001.
